# Nonlinear Optical Properties of Porphyrin, Fullerene and Ferrocene Hybrid Materials

**DOI:** 10.3390/ma14164404

**Published:** 2021-08-06

**Authors:** Francesca Limosani, Francesca Tessore, Gabriele Di Carlo, Alessandra Forni, Pietro Tagliatesta

**Affiliations:** 1 Photonics Micro and Nanostructures Laboratory, Physical Technologies for Safety and Health Division, Fusion and Technologies for Nuclear Safety and Security Department, ENEA C.R. Frascati, Via E. Fermi 45, 00044 Frascati, Rome, Italy; francesca.limosani@enea.it; 2 Department of Chemistry, University of Milan, INSTM Research Unit, Via C. Golgi 19, 20133 Milan, Italy; gabriele.dicarlo@unimi.it; 3 CNR-SCITEC, Istituto di Scienze e Tecnologie Chimiche “G. Natta”, c/o University of Milan, Via Golgi 19, 20133 Milan, Italy; alessandra.forni@scitec.cnr.it; 4 Department of Chemical Science and Technologies, University of Rome “Tor Vergata”, Via della Ricerca Scientifica 1, 00133 Rome, Italy; pietro.tagliatesta@uniroma2.it

**Keywords:** porphyrins, fullerene, ferrocene, nonlinear optics, hybrid materials, dyads, triads

## Abstract

In this research, we investigated the second-order nonlinear optical (NLO) properties of multicomponent hybrid materials formed by *meso*-tetraphenylporphyrin P (both as free base and Zn^II^ complex), carrying in 2 or 2,12 β-pyrrolic position an electron donor ferrocene (Fc), and/or an electron acceptor fullerene (C60) moiety, connected to the porphyrin core via an ethynyl or an ethynylphenyl spacer. We measured the NLO response by the electric-field-induced second-harmonic generation (EFISH) technique in CH_2_Cl_2_ solution with a 1907 nm incident wavelength, recording for all the investigated compounds unexpected negative values of μβ_1907_. Since density functional theory (DFT) calculations evidenced for P-Fc dyads almost null ground state dipole moments and very low values for P-C60 dyads and Fc-P-C60 triads, our EFISH results suggested a significant contribution to γ_EFISH_ of the purely electronic cubic term γ(−2ω; ω, ω, 0), which prevails on the quadratic dipolar orientational one μβ(−2ω; ω, ω)/5kT, as confirmed by computational evidence.

## 1. Introduction

In the last two decades, many organic and organometallic molecular chromophores have attracted attention in the scientific community for their significant second-order NLO properties, mainly arising from push–pull structures as donor—π-delocalized spacer—acceptor systems [1,2]. Among them, porphyrins and metalloporphyrins are very appealing, thanks to their thermal and chemical stability and the quite good solubility [3]. The electron-rich extended 18-electron π-conjugated core of porphyrins (P) can act as a spacer between the donor and the acceptor group in the push–pull system [4,5,6,7], or it can itself be the donor or the acceptor part of the push–pull architecture [8,9,10].

The four *meso*, the eight β-pyrrolic, and the two axial positions allow a wide variety of chemical functionalizations, so that many different substituents can be linked to the core and to the metal center. Moreover, by changing the metal center, its oxidation state, the type of the axial ligands, the nature of the substituents at the periphery of the macrocycle, the flexibility of the synthetic process and of structural diversification can be exploited to achieve a fine-tuning of the electronic properties and a large second-order NLO response [3].

Through the electric-field-induced second-harmonic generation (EFISH) technique [11,12], the effect of the metal [13], of the nature and of the position of the substituents [8,9,14], and of the presence of aggregation phenomena in solution [15,16] have been investigated in depth. Recently, also the non-negligible role of third-order contributions to the quadratic hyperpolarizability of A_4_ β-substituted Zn^II^ porphyrins was highlighted [17].

The porphyrin core has the same structure as chlorophyll and therefore has been thoroughly studied as an efficient light harvester, for example, in dye-sensitized solar cells [18,19,20,21,22,23,24] or in systems conceived to reproduce the complex electron transfer reactions occurring in natural photosynthesis. In these molecular mimetics, porphyrins were coupled at first to benzoquinone [25], then benzoquinone was replaced by fullerene (C60) [26] to originate conjugates with increased excited charge-separated state lifetime, due to a faster forward electron transfer and a slower rate for the charge recombination reaction.

In this context, C60 has been a subject of several investigations by the scientific community, because of its unique physical and chemical properties [27,28,29]. The highly symmetric structure of C60, its ability to undergo multiple addition reactions, combined with its exceptional electron-accepting characteristics (e.g., it can accept up to six electrons) are by far the most important properties [30]. With its strong electron-accepting properties and remarkably small reorganization energy (ca. 0.23 eV), C60 is one of the most popular compounds incorporated into multicomponent molecular architecture to tune its optical properties to specific spectral regions of interest. By using chemical methods, the C60 moiety, acting as an electron acceptor in the ground electronic state, significantly improves the average hyperpolarizabilities in fullerene derivatives [31]. The observed enhancement is mainly due to the delocalization of charge from the electron-rich moiety to the electron-poor carbon cage, producing partially negatively charged fullerene moieties. For this reason, chemical modifications with a variety of electron-donating organic moieties have been studied [32].

Many other porphyrin–C60 dyads (P-C60) have been reported [33], with C60 linked through an amide bridge to the porphyrin *meso*-phenyl ring [34] or to the β-pyrrolic position of the core in various fashion [35,36,37].

The continuous interest in the realization of complex architectures with a long lifetime of charge-separated state has led research groups to perform extensive studies on different linear multi-porphyrin–fullerene adducts, some equipped with an additional moiety, mainly ferrocene (Fc) [38,39,40,41,42]. Because of the low oxidation potential of the iron atom, Fc displays electron-donating properties [43].

The properties of P-C60, P-Fc or Fc-P-C60 conjugates can be modified by changing the binding position (*meso* or β-pyrrolic) of Fc and/or C60 on the porphyrin ring or the type of connection (double or triple bond). For example, when Fc and C60 are covalently linked to the phenyl groups of a Zn^II^ complex of a *meso*-tetraphenylporphyrin, as in Fc-NHCO-ZnP-NHCO-C60, a charge separation lifetime of 8 µs is observed [33], which becomes two orders of magnitude higher when Fc and C60 are connected to the porphyrin core by imidazole linkers through the β,β’-pyrrolic positions [44].

Binding of Fc moiety to the β-pyrrolic position of P through an ethynyl group is an effective way to enhance the electronic interaction between the π-system of the macrocycle and the organometallic moiety [45]. On the other hand, the introduction of an additional phenyl spacer between Fc and P provides only a small effect, as revealed by both spectroscopic and electrochemical measurements [45].

The connection of C60 to the β-pyrrolic position of P by an ethynylphenyl bridge produces an efficient donor–acceptor system, where the lowest unoccupied and the highest occupied molecular orbitals (LUMO and HOMO) are localized on P and on C60, respectively [37].

In Fc-P-C60 triads a high charge delocalization of the π-electrons between the donor Fc and the acceptor C60 moieties is reached by linking them to the 2,12 β-pyrrolic positions of P through an ethynyl or an ethynylphenyl spacer [46], thus constituting an efficient approach to the modulation of electron donor–acceptor interactions to realize hybrid materials [47,48,49,50,51,52].

Since the linkage of electron donor Fc and/or electron acceptor C60 to P turns out in Fc-P and C60-P dyads and Fc-P-C60 triads to have a push–pull structure, they might display interesting second–order NLO properties.

**Figure 1:**
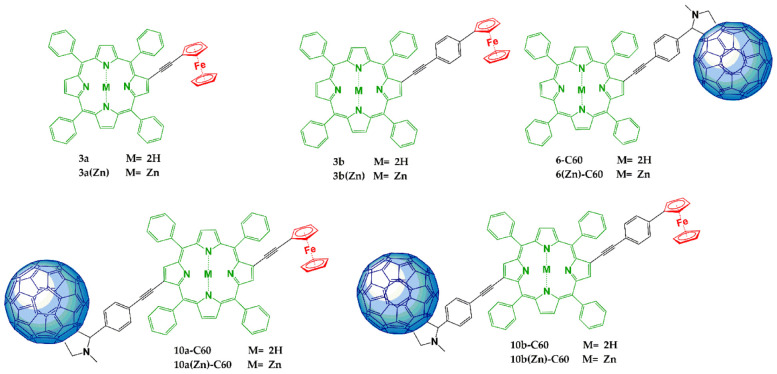
Investigated push–pull hybrid materials.

Therefore, this work aims to report and discuss the results of an EFISH investigation of the dyads and triads reported in Figure 1, which have never been investigated from a nonlinear optical point of view.

## 2. Materials and Methods

### 2.1. Materials

Dyads **3a**, **3b**, **3a(Zn)**, **3b(Zn)** were synthesized and characterized as reported in [53]. Dyads **6-C60** and **6(Zn)-C60** were synthetized and characterized as reported in [36]. Triads **10a-C60, 10a(Zn)-C60, 10b-C60, 10b(Zn)-C60** were synthetized and characterized as reported in [46]. Their NMR, MS and UV spectra were in accordance with those previously reported (see Supplementary Materials, Figures S1–S6 and S9–S16, and the elemental analyses).

Electronic absorption spectra of compounds **6-C60** and **6(Zn)-C60** in CH_2_Cl_2_ solution were recorded at room temperature on a Shimadzu UV 3600 spectrophotometer (Shimadzu Corporation, Kyoto, Japan) and are reported in the Supplementary Materials (Figures S7 and S8).

### 2.2. EFISH Measurements

EFISH experiments were performed on freshly prepared 10^−3^ M CH_2_Cl_2_ solutions. The incident wavelength at 1.907 µm was obtained by Raman shifting the 1.064 µm emission of a Q-switched Nd:YAG laser in a high-pressure hydrogen cell (60 bar). The 1.907 μm laser incident wavelength was chosen because its second harmonic (at 953 nm) was far enough from the absorption bands of the chromophores in CH_2_Cl_2_ [46] to avoid any enhancement of the second-order NLO response because of resonance. The Maker fringe pattern (that is the harmonic intensity variation as a function of the cell translation) was obtained through a liquid cell with thick windows in the wedge configuration. In the EFISH experiments, the incident beam was synchronized with a DC field applied to the solution, with 60 and 20 ns pulse durations, respectively, to break its centrosymmetry. The NLO response (assumed to be real because the imaginary part was neglected) was determined from the concentration dependence of the harmonic signal with respect to that of the pure solvent through the experimental value γ_EFISH_ Equation (1):

(1) $$\gamma_{\mathrm{EFISH}}=\frac{{\mu \beta }_{\lambda }\left(-2\omega ;\omega ,\omega \right)}{5\mathrm{kT}}+\gamma \left(-2\omega ;\omega ,\omega ,0\right).$$

γ_EFISH_ is the sum of the purely electronic cubic contribution γ(−2ω; ω, ω, 0) and of a quadratic dipolar orientational contribution μβ_λ_(−2ω; ω, ω)/5kT, μ being the ground state dipole moment, and β_λ_ the projection along the dipole moment direction of the vectorial component β_vec_ of the tensorial quadratic hyperpolarizability working with the incident wavelength λ.

The EFISH experiments were performed recording firstly the second-order response of the pure solvent, then the second-order response of the chromophore in solution, and finally the second-order response of the solvent again. The EFISH values reported were the average of 12 consecutive measurements performed on the same sample. The uncertainty of the measure was about ±15%.

All the experimental EFISH β_1.907_ values were defined according to the “phenomenological” convention [54].

The apparatus for the EFISH measurements was a prototype made by SOPRA (Paris, France) and the experiments were carried out in the Department of Chemistry of the University of Milano (Italy).

### 2.3. Computational Details

Density functional theory (DFT) calculations were performed on all compounds using the Gaussian16 suite of programs [55]. Geometry optimizations were performed with the 6-311G(d) basis set using the PBE0 functional [56,57] in CH_2_Cl_2_, adopting the polarized continuum model in its integral equation formalism (IEFPCM) to describe the solvent effect [58]. Using the same basis set, the SHG first hyperpolarizabilities, i.e., the β(−2ω; ω, ω) tensors, were computed within the coupled perturbed Kohn−Sham (CPKS) approach at the same frequency (1907 nm) used in the EFISH experiments. The SHG second hyperpolarizabilities, i.e., the γ(−2ω; ω, ω, 0) tensors, were evaluated by finite field technique. β and γ calculations were performed by using the M06-2X functional [59], owing to its optimal performance in reproducing hyperpolarizability values for midsize chromophores [60]. A pruned (99,590) grid was selected for computation and use of two-electron integrals and their derivatives. From the full tensors **β** and **γ**, the scalar quantities β_||_ and γ_||_, respectively, were derived to get a meaningful comparison with the experimental data. β_||_ corresponds to 3/5 times β_λ_, the projection along the dipole moment direction of the vectorial component of the β tensor, that is, β_||_ = (3/5) ∑_i_(μ_i_β_i_)/μ, where β_i_ = (1/5)∑_j_(β_ijj_ + β_jij_ + β_jji_) [61,62].

γ_||_ is related to the tensor components according to the following: γ_||_ = (1/15) [3(γ_xxxx_ + γ_yyyy_ + γ_zzzz_) + 2(γ_xxyy_ + γ_xxzz_ + γ_yyzz_ + γ_yyxx_ + γ_zzxx_ + γ_zzyy_) + (γ_xyyx_ + γ_xzzx_ + γ_yzzy_ + γ_yxxy_ + γ_zxxz_ + γ_zyyz_)] [61].

## 3. Results and Discussion

### 3.1. Synthesis

The dyads and triads investigated in this work have never been considered for nonlinear optics. They have been synthesized and characterized according to the literature, as highlighted in Section 2.1 [36,46,53].

**Scheme 1:**
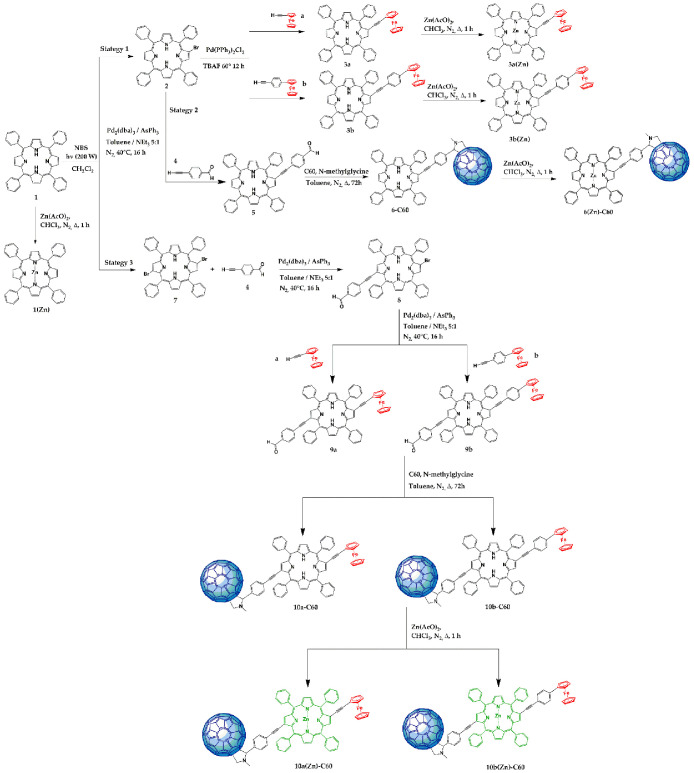
Different synthetic routes toward push–pull hybrid materials.

However, since their preparation was not trivial, we summarized the main synthetic details (Scheme 1).

The possibility of placing different substituents at the β-pyrrole positions constituted a powerful approach toward the fine-tuning of tetrapyrroles and the modulation of electron donor–acceptor interactions to realize performing hybrid materials.

In Scheme 1 we show three different synthetic strategies for creating push–pull systems formed by a combination of electron donors (i.e., ferrocene), light harvester (i.e., porphyrin), and electron acceptors (i.e., C60) connected to each other through “molecular wires” of variable lengths.

Specifically, ethynyl or ethynylphenyl functionalities were selected as molecular bridges because of their synthetic versatility and their outstanding physicochemical properties. It was previously reported that these linkers assist in a good conduction of the charges due to their high electron density and the extended π-system [63,64,65].

The use of synthetic approaches that involved the Sonogashira reaction or its modification for the formation of carbon–carbon bonds allowed obtaining final mono and disubstituted compounds in β-positions of the macrocycle.

The first step involved the bromination of compound **1** using different quantities of N-bromosuccinimide (NBS) to obtain preferentially monobromo-(**2**) and dibromo-porphyrin (**7**). The bromination of specific antipodal pyrrole position 2 and 2,12 was carried out following the procedure from the literature [66], using light-induced reaction and NBS in CH_2_Cl_2_. The first synthetic strategy (Strategy 1) consisted of a variation of Sonogashira coupling introduced by Li and coworkers [67] for the formation of monosubstituted compounds by linking different donor units.

Specifically, we used ferrocenyl moieties of variable length such as ethynylferrocene (**a**) or 4-(ferrocenyl)-phenylacetylene (**b**) to functionalize the β positions of the macrocycle in one step.

The approach was based on the use of tetrabutylammonium fluoride (TBAF) as reagent under copper-, amine-, and solvent-free conditions. We obtained the final compounds **3a** and **3b** in 50–65% of yield [53], starting from compound **2** and using Pd(PPh_3_)_2_Cl_2_ as catalyst.

The second strategy (Strategy 2) involved a Sonogashira coupling, using the catalytic system Pd_2_(dba)_3_/AsPh_3_ developed by Lindsey and coworkers [68,69,70], paying particular attention to the deoxygenation and dilution conditions and, most of all, avoiding the use of copper iodide as cocatalyst.

In this way the homocoupling side reaction between terminal alkynes was suppressed, and the desired final hybrid materials were formed by the combination of porphyrin and fullerene as acceptor unit.

In this synthetic strategy the Sonogashira coupling of compound **2** with 1.5 equivalents of *p*-ethynylbenzaldehyde (**4**) afforded porphyrin **5**. For the functionalization of C60 with intermediate **5**, we used the Prato–Maggini reaction [71] to achieve the final compound **6-C60** in 60% of yield [36].

To obtain disubstituted hybrid materials formed in the same structure by an acceptor and donor units, the third synthetic strategy was adopted (Strategy 3).

In addition, in this case the first step involved a Sonogashira coupling of compound **7** with 1.5 equivalents of compound **4** to obtain compound **8**.

The next step was again a Sonogashira coupling reaction between compound **8** and two equivalents of different ferrocene units (**a**-**b**) to afford the corresponding intermediates **9a** and **9b**.

The obtained compounds **9a** and **9b** were subsequently used for the cycloaddition reaction with **C60** [63], affording the desired **10a-C60** and **10b-C60** systems in 41 and 35% of yield, respectively [46].

In the final step of the different synthetic strategies, the respective Zn^II^ porphyrinate complexes of all compounds (**1**, **3a, 3b, 6-C60**, **10a-C60**, **10b-C60**) were obtained by dissolving the compounds in chloroform and adding a slight excess of a saturated Zn(OAc)_2_ methanol solution to yield **1(Zn)**, **3a(Zn), 3b(Zn), 6(Zn)-C60, 10a(Zn)-C60** and **10b(Zn)-C60** quantitatively.

### 3.2. UV-Vis Spectroscopy

The electronic properties of the dyads and triads were investigated by UV-Vis spectroscopy in CH_2_Cl_2_ solution (Table 1). While the spectra of **6-C60** and **6(Zn)-C60** were recorded for the first time (Figures S7 and S8 in the Supplementary Materials), those of the other compounds have already been reported [37,46,53].

The UV-Vis spectra of the free-base porphyrins (**3a**, **3b**, **6-C60**, **10a-C60** and **10b-C60**) and of their Zn^II^ complexes (**3a(Zn)**, **3b(Zn)**, **6(Zn)-C60**, **10a(Zn)-C60** and **10b(Zn)-C60**) fulfilled the “four orbital model” developed by Gouterman [72]. The S_0_➔S_2_ (ground➔second excited state) transition produced the intense (logε in the range 4.50–5.43) Soret or B band at 420–450 nm, and the S_0_➔S_1_ (ground➔first excited state) transitions led to four (for free bases) or two (for the Zn^II^ complexes) weaker (logε in the range 3.42–4.50) Q bands at 520–670 nm. The reduction of the number of the Q bands by complexation was because of the increased degree of microsymmetry, from *D*_2h_ of the free base to *D*_4h_ of the metal complex [72].

The complexation to the metal ion induced a 3–15 nm bathochromic shift of the B band. A 2 nm redshift by complexation occurred also for the Q_III_ band of dyads **3b** and **6-C60**. On the other hand, by complexation to Zn^II^ of triads **10a-C60** and **10b-C60** the Q_III_ band underwent a 4–6 nm ipsochromic shift and increased in intensity.

The UV-Vis data allowed us to highlight the effect that the introduction of Fc and/or C60 may have had on the electronic properties of P (and of its Zn^II^ complex) [73].

Starting from 5, 10, 15, 20-tetraphenylporphyrin **1** (Scheme 1), the introduction in β-pyrrolic position of an electron-donating Fc moiety connected to the core by a triple bond (**3a**) produced a sizable redshift of the B and the Q bands (9 and 11–18 nm, respectively), suggesting an increased molecular conjugation. Conversely, the introduction in **3a** of an additional phenyl unit between Fc and P (**3b**) slightly affected the spectroscopic properties: the B and Q_IV_ bands were almost the same, while the other three Q bands experienced a slight ipsochromic shift (1–3 nm). Hence, the insertion of the phenyl moiety was not effective in further enhancing π-delocalization.

Furthermore, linking an electron-withdrawing C60 moiety to **1** by an ethynylphenyl spacer (**6-C60**) led to a significant redshift of the B and Q bands (10 and 7–11 nm, respectively) and to an increased conjugation. However, different from what was observed when a –NO_2_ [17] or a cyanoacrylic moiety [24,65,74] was connected in the same fashion to a Zn^II^-porphyrin, the B band of **6(Zn)-C60** was symmetric, without any shoulder at lower energy.

In the spectra of **6-C60** and **6(Zn)-C60** the well-defined contribution of the C60 unit was also present at 255 and 329 nm, respectively [75].

Therefore, the insertion of a Fc or a C60 moiety on P affects and tunes its electronic properties, promoting a charge transfer process from the π-conjugated substituent in β-pyrrolic position to the macrocycle when the former carries an electron donor, and from the macrocycle to the π-conjugated system when this latter has an electron acceptor [17]. In other words, P behaves as an electron acceptor moiety when connected to electron-rich Fc and as an electron donor when connected to electron acceptor C60, displaying an ambivalent role [8,10].

When both Fc and C60 were bound to P in 2,12 β-pyrrolic positions [76] (triads **10a-C60** and **10b-C60** and their Zn^II^ complexes), the UV-Vis data showed a further redshift of the B and Q bands, which was more significant for the first triad.

### 3.3. EFISH Investigation of the Second-Order NLO Properties

We measured the second-order NLO response of our compounds by the EFISH technique on 10^−3^ M CH_2_Cl_2_ solutions with a 1907 nm incident wavelength. The details are in the Materials and Methods Sections, and the results in Table 2.

**Figure 2:**
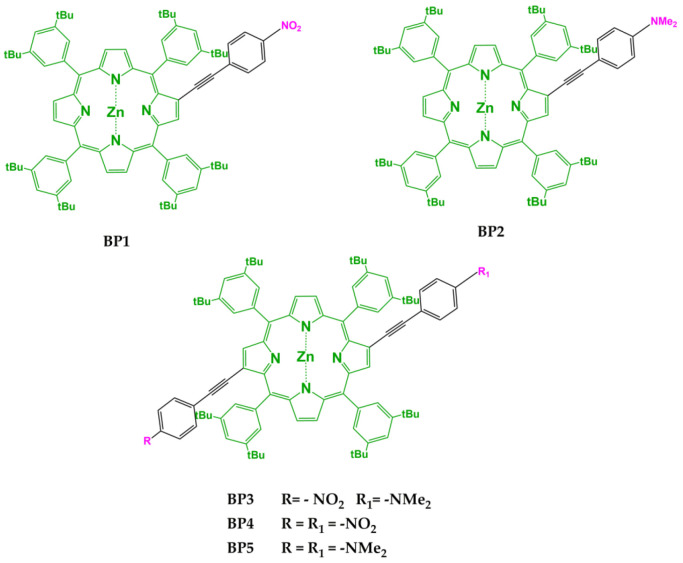
A_4_ β-pyrrolic Zn^II^ porphyrins similar to the ones here investigated.

All the investigated compounds showed negative γ_EFISH_ and µβ_1907_ values. For dyads **6-C60** and **6(Zn)-C60** and for triads **10a(Zn)-C60** and **10b(Zn)-C60** this outcome was quite unexpected. Indeed, the similar complexes BP1 and BP3 (Figure 2), with a –NO_2_ acceptor group (instead of C60) and a –NMe_2_ donor group (instead of Fc) linked to the core by an ethynylphenyl moiety in 2 and 2,12 β-pyrrolic position, displayed positive γ_EFISH_ and μβ_1907_ values [17].

On the other hand, the negative γ_EFISH_ and μβ_1907_ of dyads **3a**, **3a(Zn)**, **3b,** and **3b(Zn)** were in agreement with what was observed for BP2, where Fc was replaced by –NMe_2_ [17].

According to the “two-level” model developed by Oudar [77,78], a negative sign of μβ_λ_ derives from a negative value of Δμ_eg_, which is the difference between the excited and the ground state molecular dipole moments. Negative μβ_λ_ values suggest a decrease of the excited state dipole moment in comparison to the ground state [79]. This evidence occurred for C60 containing second-order NLO chromophores, in which the C60 acceptor moiety endows a cyclopropane ring bridging an ethynylthienyl spacer linked to a trimethylsilyl or an alkynyl platinum donor unit [80].

Moreover, when the second-order NLO response obtained by the EFISH technique showed an unexpected sign and/or absolute value of β_λ_, aggregation or other molecular interactions occurring in solution should be considered [15,16].

Nevertheless, A_4_ β-pyrrolic mono or disubstituted Zn^II^ porphyrins were characterized by a remarkable steric hindrance, because of the 70–90° dihedral angle formed by the aryl rings in 5, 10, 15, 20 *meso* position with the mean plane of the macrocycle, which lowered the flatness of the molecule and hampered aggregation phenomena in solution [16,76].

The μβ_1907_ values in Table 2 derive from Equation (1), neglecting the cubic electronic contribution γ_0_(−2ω; ω, ω, 0) to γ_EFISH,_ and for this reason could be overestimated. Indeed, the EFISH technique is appropriate to study dipolar chromophores with a clear push–pull structure, for which the third-order contribution is much smaller than the quadratic dipolar orientational term and can be neglected. However, for macrocycles with an extended π-conjugation and significant third-order NLO properties (asymmetrically monosubstituted metal porphyrins [81], phtalocyanines [82] or porphyrazines [83]), the EFISH second-order NLO response could be affected by a significant error, since the cubic term is comparable, at least as an order of magnitude, to the quadratic orientational one. Moreover, as recently reported by some of us for the BP2 complex (Figure 2) [17], when the molecular ground state dipole moment (μ) is low, the electronic third-order term can have an overwhelming role, determining the sign of the second-order response.

To clarify these aspects, we computed the ground state dipole moments of our dyads and triads by DFT (Table 2).

The µ values for all the P-Fc dyads (**3a**, **3a(Zn)**, **3b** and **3b(Zn)**) were almost null, suggesting for these compounds a very low dipolar character. Notably, **3a(Zn)** and **3b(Zn)** showed the lowest μ ever computed for A_4_ β-pyrrolic monosubstituted Zn^II^ porphyrins [8,17], with values comparable in the order of magnitude only to the ones reported for symmetric structures such as BP4 and BP5 (Figure 2). Therefore, endowing the porphyrin core either as a free core or as a Zn^II^ complex with a donor Fc moiety did not produce an efficient push–pull system, despite the increased molecular conjugation pointed out by the UV-Vis spectroscopic data (Table 1). In other words, the electronic perturbation induced by Fc in the β-pyrrolic position of the macrocycle was trivial. The overall polarizability of the system increased, but without any sizable asymmetry in the electronic density distribution.

Hence, we can safely conclude that, similar to what was reported for slightly asymmetric BP2 and for symmetric BP4 and BP5 [17], in P-Fc dyads the electronic third-order contribution γ_0_(−2ω; ω, ω, 0) to γ_EFISH_ outstrips the dipolar orientational term µβ_1907_(−2ω; ω, ω)/5kT. Basically P-Fc dyads behave as third-order NLO chromophores, because of their almost null polarity.

This conclusion is supported by CP-DFT calculations in dichloromethane, which we chose to perform only on the Zn^II^ dyads and triads similar to BP1, BP2 and BP3, as the most representative of our series (Table 3).

As expected, **3b(Zn)** showed a very low β_||_ value as a consequence of a negligible μβ_||_/5kT in comparison to the high and negative γ_||_ (Table 3).

In contrast, linking a C60 moiety to P produced dyads (**6-C60** and **6(Zn)-C60**) with μ values in the range 3.5–4.8 D, in agreement with a decrease of the electron density on P when connected to acceptor C60 and playing the role of the electron donor part of the push–pull system [8]. The acceptor character of C60 appeared lower than that of the –NO_2_ group, since for BP1 a μ value of 7.8 D was computed (compared with 4.77 of **6(Zn)-C60**) [17]. Moreover, an enhancement of μ occurred by complexation (the μ of **6-C60** was 4.21 D and that of **6(Zn)-C60** 4.77 D) [8].

Nevertheless, P-C60 dyads, albeit more polar than the P-Fc counterparts, still showed a low molecular asymmetry. In agreement with the enhanced μ value and the more pronounced push–pull character, the computed β_||_ and μβ_||_/5kT of **6(Zn)-C60** were higher than those of **3b(Zn)**, but the γ_EFISH_ was still dominated by the third-order contribution, which produced a negative sign.

Eventually, the μ values of the triads were similar one to the other and to those of P-C60 dyads, confirming the insignificant contribution of the Fc unit to the polarity of the system, even in the presence of the additional phenyl spacer between Fc and P. An increase of μ occurred by complexation (3.82 D vs. 4.37 D for **10a-C60** and **10a(Zn)-C60** and 3.82 D vs. 4.14 D for **10b-C60** and **10b(Zn)-C60**). Once again, the computed β_||_, μβ_||_/5kT and γ_||_ values supported an overwhelming contribution of the cubic term to γ_EFISH_. Moreover, the absolute value of γ_||_ of **10b(Zn)-C60** was the highest among the series (Table 3), since the introduction of the ethynylphenyl spacer carrying the Fc moiety extended π-delocalization, as evidenced by the bathochromic shift of the B and Q bands in the UV-Vis spectra (Table 1).

## 4. Conclusions

In this work, we investigated the second-order NLO properties of a series of dyads and triads composed by *meso*-tetraphenylporphyrin P (both as free base and Zn^II^ complex), carrying in 2 or 2,12 β-pyrrolic position an electron donor ferrocene (Fc), and/or an electron acceptor fullerene (C60) moiety, connected to the porphyrinic core via an ethynyl or an ethynylphenyl spacer.

UV-Vis spectroscopy showed that the introduction of a Fc or a C60 unit on the P core causes a sizable bathochromic shift of the B and Q bands of the tetrapyrrolic macrocycle, pointing to an increased molecular conjugation, in particular, with an ethynyl spacer. Indeed, the insertion of an additional phenyl moiety between Fc and P did not enhance π-delocalization significantly. On the other hand, linking both Fc and C60 to P produced a further redshift of the electronic absorption bands.

Therefore, the presence of Fc and /or C60 tuned the electronic properties of P in such a way that it behaved as an electron acceptor when connected to electron-rich Fc and as an electron donor when connected to electron-deficient C60, confirming its ambivalent role [8,10].

Surprisingly, EFISH measurements produced for all the investigated compounds negative γ_EFISH_ and µβ_1907_ values. Since A_4_ β-pyrrolic mono or disubstituted Zn^II^ porphyrins feature a sterically hindered architecture [16,76], we could safely exclude the presence of aggregation phenomena in solution, which could affect the sign and magnitude of the EFISH response.

However, when the ground state molecular dipole moment was low, the pure electronic cubic contribution to γ_EFISH_ γ_0_(−2ω; ω, ω, 0) overwhelmed the dipolar orientational term μβ_1907_(−2ω; ω, ω)/5kT, dictating the sign of the second-order response [17].

DFT-computed dipole moments of P-Fc dyads were almost null, and for P-C60 dyads and Fc-P-C60 triads they were in the range 3.5–4.8 D, suggesting for all the investigated compounds a low polarity, which led to a non-negligible third-order contribution to their second-order NLO response, as confirmed by the calculated β_||_, μβ_||_/5kT and γ_||_ values of representative dyads and triads.

Therefore, our investigation proved from both an experimental and a theoretical point of view that the combination of porphyrins, fullerene, and ferrocene leads to hybrid materials with a high polarizability but a low push–pull character, whose second-order NLO properties as measured by the EFISH technique must be analyzed very carefully.

## Figures and Tables

**Table 1:** Synopsis of the UV-Vis spectroscopic data in CH_2_Cl_2_ of the investigated dyads and triads.

Compound	Soret Band λ_max_ (nm) (logε)	Q_IV_ Band λ_max_ (nm) (logε)	Q_III_ Band λ_max_ (nm) (logε)	Q_II_ Band λ_max_ (nm) (logε)	Q_I_ Band λ_max_ (nm) (logε)
**3a**	426 (5.30)	526 (4.22)	566 (3.93)	602 (3.95)	660 (3.56)
**3a(Zn)**	432 (4.50)		566 (4.08)	602 (3.83)	
**3b**	427 (5.28)	526 (4.29)	563 (3.94)	601 (3.79)	658 (3.51)
**3b(Zn)**	436 (5.43)		565 (4.37)	601 (4.06)	
**6-C60**	427 (5.31)	522 (4.27)	558 (3.83)	599 (3.76)	656 (3.42)
**6(Zn)-C60**	434 (5.28)		560 (4.15)	598 (4.00)	
**10a-C60**	434 (5.15)	527 (4.59)	580 (3.81)	616 (3.61)	670 (3.71)
**10a(Zn)-C60**	449 (5.36)		574 (4.37)	613 (4.34)	
**10b-C60**	435 (5.23)	532 (4.37)	574 (4.21)	609 (4.00)	666 (3.68)
**10b(Zn)-C60**	438 (5.20)		570 (3.76)	612 (3.61)	
**1**	417 (5.58)	515 (4.19)	550 (3.83)	591 (3.68)	647 (3.61)
**1(Zn)**	420 (5.78)		548 (4.41)	589 (3.76)	

**Table 2:** Experimental γ_EFISH_ and μβ_1907_ values (10^−3^ M solution in CH_2_Cl_2_) and theoretical dipole moments (μ) for the investigated dyads and triads.

Compound	μ (D)	γ_EFISH_ (x × 10^−33^ esu)	µβ_1907_ (x × 10^−48^ esu)
**3a**	0.09	−1.54	−320
**3a(Zn)**	0.19	−3.11	−640
**3b**	0.49	−1.88	−390
**3b(Zn)**	0.28	−2.85	−595
**6-C60**	4.21	−2.84	−590
**6(Zn)-C60**	4.77	−3.47	−720
**10a-C60**	3.82	−7.12	−1495
**10a(Zn)-C60**	4.37	−8.08	−1670
**10b-C60**	3.82	−5.19	−1075
**10b(Zn)-C60**	4.14	−6.41	−1330

**Table 3:** Theoretical β_||_, μβ_||_/5kT and γ_||_ values of representative dyads and triads.

Compound	β_||_ (x × 10^−30^ esu)	μβ_||_/5kT (x × 10^−36^ esu)	γ_||_ (x × 10^−^^36^ esu)
**3b(Zn)**	−21	−29	−1820
**6(Zn)-C60**	30	696	−1543
**10b(Zn)-C60**	42	845	−3225

## Supplementary Materials

The following are available online at https://www.mdpi.com/article/10.3390/ma14164404/s1, Figure S1: ^1^H-NMR spectrum of compound **3a** in CDCl_3_ at 300 K at 400 MHz, Figure S2: ^1^H-NMR spectrum of compound **3a(Zn)** in CDCl_3_ at 300 K at 400 MHz, Figure S3: ^1^H-NMR spectrum of compound **3b** in CDCl_3_ at 300 K at 400 MHz, Figure S4: ^1^H-NMR spectrum of compound **3b(Zn)** in CDCl_3_ at 300 K at 400 MHz, Figure S5: ^1^H-NMR spectrum of compound **6-C60** in CDCl_3_ at 300 K at 400 MHz, Figure S6: FAB MS spectrum of compound **6-C60** using as matrix NBA, Figure S7: UV-Vis spectrum of compound **6-C60** in CH_2_Cl_2_, Figure S8: UV-Vis spectrum of compound **6(Zn)-C60** in CH_2_Cl_2_, Figure S9: ^1^H-NMR spectrum of compound **10a-C60** in CDCl_3_ at 300 K at 400 MHz, Figure S10: MALDI-TOF MS spectrum of compound **10a-C60** using as matrix gentisic acid, Figure S11: ^1^H-NMR spectrum of compound **10a(Zn)-C60** in CDCl_3_ at 300 K at 400 MHz, Figure S12: MALDI-TOF MS spectrum of compound **10a-C60** using as matrix gentisic acid, Figure S13: ^1^H-NMR spectrum of compound **10b-C60** in CDCl_3_ at 300 K at 400 MHz, Figure S14: MALDI-TOF MS spectrum of compound **10b-C60** using as matrix gentisic acid, Figure S15: ^1^H-NMR spectrum of compound **10b(Zn)-C60** in CDCl_3_ at 300 K at 400 MHz, Figure S16: MALDI-TOF MS spectrum of compound **10b(Zn)-C60** using as matrix gentisic acid.
