# Supplementary material for: Nonlinear Optical Properties of Porphyrin, Fullerene and Ferrocene Hybrid Materials
Source: Materials (Basel). 2021 Aug 6;14(16):4404. doi: 10.3390/ma14164404 (PMC8401996; doi:10.3390/ma14164404)
Supplement: Supplementary file 1 [file materials-14-04404-s001.zip › materials-1316899-supplementary.pdf]

materials

MDPI

Article

Supplementary Materials: Nonlinear optical properties of porphyrin, fullerene and ferrocene hybrid materials

Francesca Limosani [1], Francesca Tessore [2,*], Gabriele Di Carlo [2], Alessandra Forni [3] and Pietro Tagliatesta[4]

[1] Photonics Micro and Nanostructures Laboratory, Physical Technologies for Safety and Health Division , Fusion and Technologies for Nuclear Safety and Security Department, ENEA C.R. Frascati, Via E. Fermi 45, Frascati, 00044 Rome, Italy; francesca.limosani@enea.it

[2] Department of Chemistry, University of Milan, and INSTM Research Unit, Via C. Golgi 19, 20133 Milan, Italy; gabriele.dicarlo@unimi.it

[3] CNR-SCITEC, Istituto di Scienze e Tecnologie Chimiche "G. Natta", c/o University of Milan, Via Golgi 19, 20133 Milan, Italy; alessandra.forni@scitec.cnr.it

[4] Department of Chemical Science and Technologies, University of Rome "Tor Vergata", Via della Ricerca Scientifica 1, 00133 Rome, Italy; pietro.tagliatesta@uniroma2.it

* Correspondence: francesca.tessore@unimi.it; Tel.: +39-0250314398

Experimental

General Methods. ^{1}H-NMR spectra were recorded as $CDCl_3$ solutions on a Bruker AM-300 instrument using residual solvent signal as an internal standard. Chemical shifts are given as δ values. FAB mass spectra were measured on a VG-4 spectrometer using m-nitrobenzyl alcohol (NBA) as a matrix. Matrix-assisted laser desorption/ionization time of flight (MALDI-TOF) mass spectra were performed with a MALDI-TOF Reflex IV instrument (Bruker-Daltonics) in reflector mode, using a 337 nm nitrogen laser (8 Hz). A 2 mg/mL 2,5-dihydroxybenzoic acid (gentisic acid) solution in CH_3CN/TFA (0.1% solution) was used as a matrix. Electronic absorption spectra were recorded in CH_2Cl_2 solution at room temperature on a Shimadzu UV 3600 spectrophotometer (Shimadzu Corporation, Kyoto, Japan).

Elemental analyses were carried out with a PerkinElmer CHN 2400 instrument in the Analytical Laboratories of the Department of Chemistry at the University of Milan.

Compound 3a

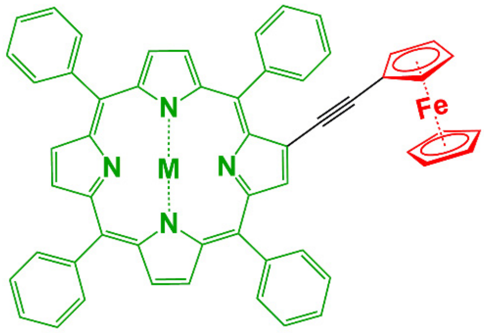

3a M= 2H

^{1}H-NMR (300 MHz, $CDCl_3$): δ(ppm) 9.02(s, 1H), 8.87(s, 2H), 8.82(m, 3H), 8.73 (m, 1H), 8.24(m, 8H), 7.83(s, 12H), 4.38(s, 3H), 4.27(s, 6H), -2.66(s, 2H).

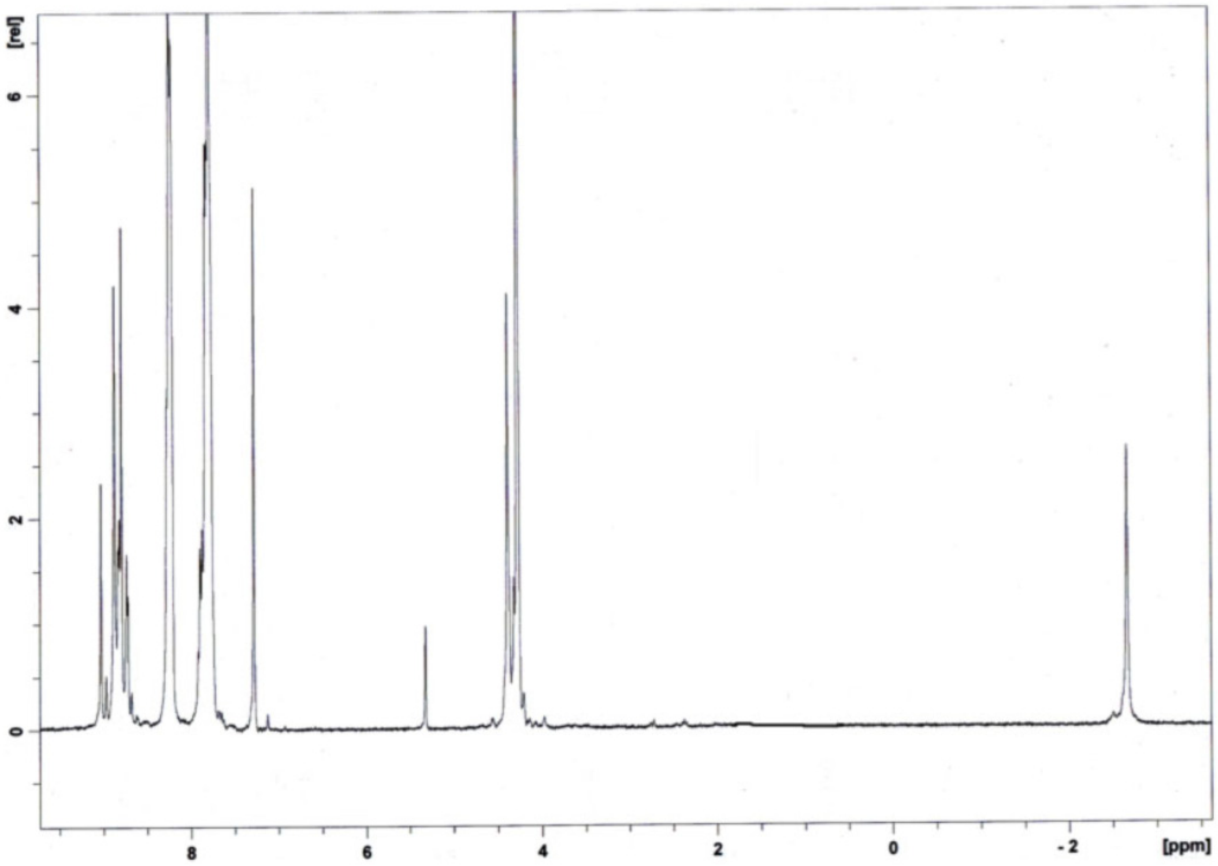

Figure S1. ^{1}H-NMR spectrum of compound **3a** in $CDCl_3$ at 300 K at 400 MHz.

MS(FAB+): *m/z:* 823 $[M+H]^+$

Elemental Analysis

Anal. Calcd. for $C_{56}H_{38}N_4Fe$: C, 81.74; H, 4.65; N,6.80. Found: C, 81.99; H, 4.64; N, 6.78

Compound 3a(Zn)

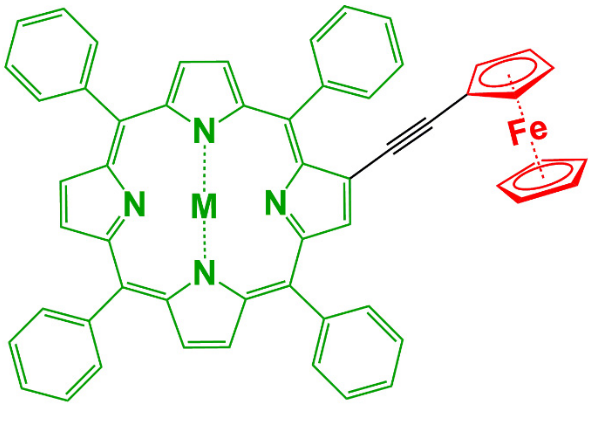

3a(Zn) M= Zn

^{1}H-NMR (300 MHz, $CDCl_3$): δ(ppm) 9.18(s, 1H), 8.92(m, 4H), 8.82(d, 2H, J=7.1 Hz), 8.75(d, 2H, J=7.1 Hz), 8.24(m, 8H), 7.80(m, 12H), 4.68(s, 1H), 4.42(s, 2H), 4.25(s, 6H).

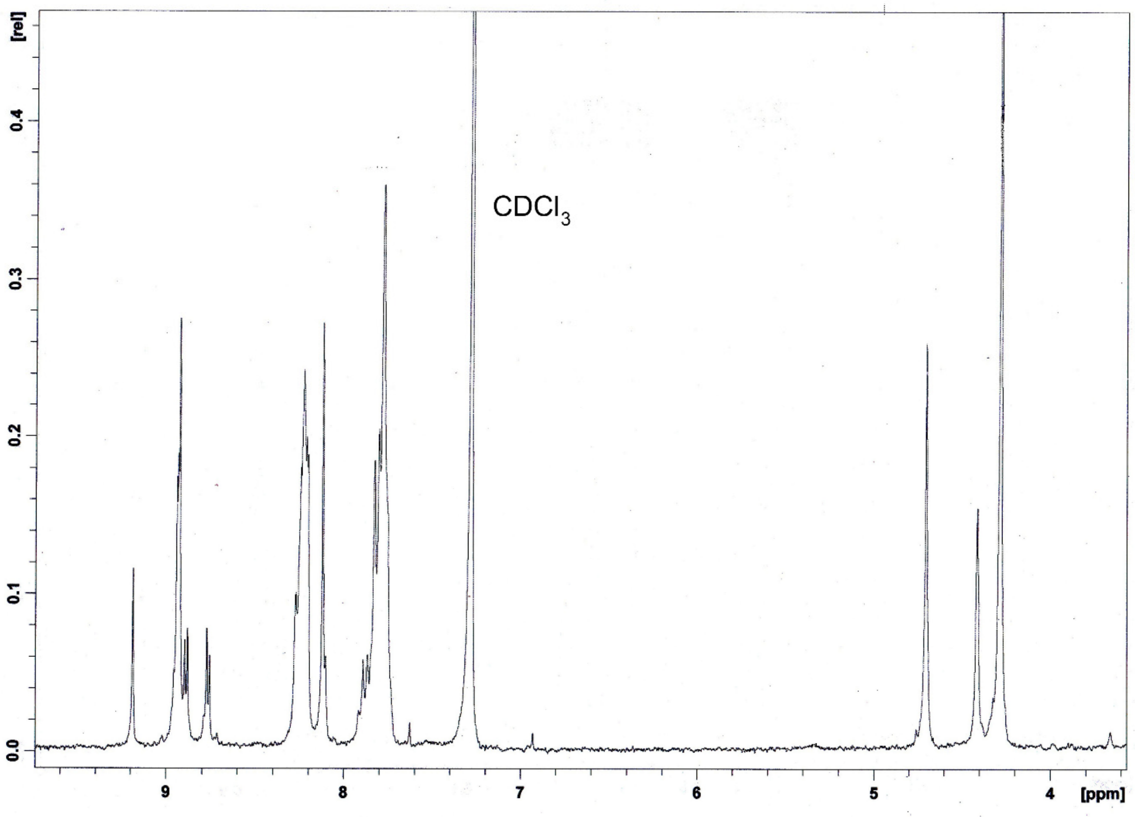

Figure S2. ^{1}H-NMR spectrum of compound **3a(Zn)** in $CDCl_3$ at 300 K at 400 MHz. .

MS(FAB+): *m*/*z*: 886 $[M]^+$

Elemental Analysis

Anal. Calcd. for $C_{56}H_{36}N_4FeZn$: C, 75.90; H, 4.09; N, 6.32. Found: C, 75.67; H, 4.08; N, 6.34

Compound 3b

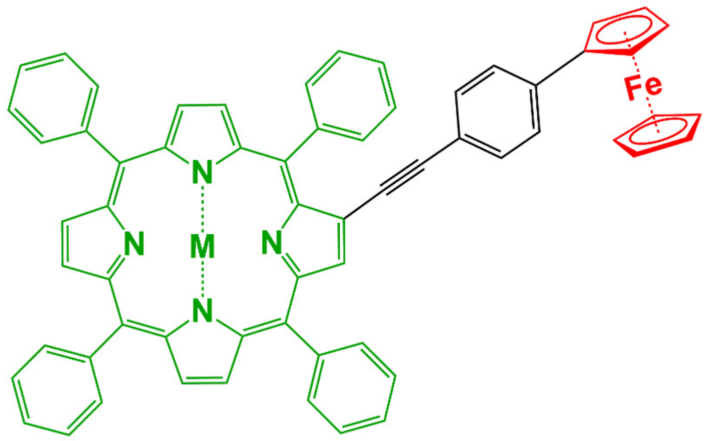

3b **M= 2H**

^{1}H-NMR (300 MHz, $CDCl_3$): δ(ppm) 9.09(s,1H), 8.87(s, 2H), 8.82(d, 2H), 8.73(m, 2H), 8.24(m, 8H), 7.83(m, 12H), 7.42(d, 2H, J=8.3 Hz), 7.29(d, 2H, J=8.3 Hz) 4.65(s, 2H), 4.38(s, 2H), 4.27(s, 5H), -2.66(s, 2H).

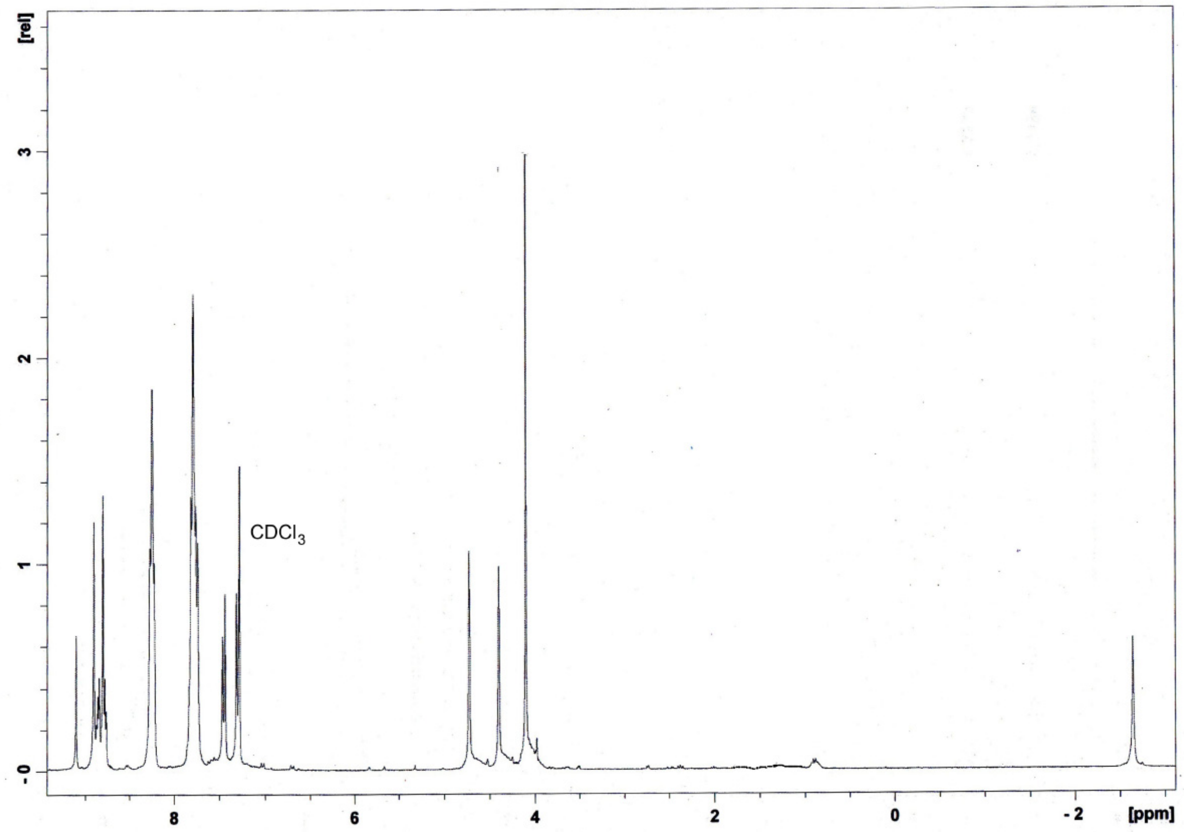

Figure S3. ^{1}H-NMR spectrum of compound **3b** in $CDCl_3$ at 300 K at 400 MHz. .

MS(FAB+): *m/z* : 899 $[M+H]^+$

Elemental Analysis

Anal. Calcd. for $C_{62}H_{42}N_4Fe$: C, 82.84; H, 4.70; N, 6.23. Found: C, 83.09; H, 4.71; N, 6.25

Compound 3b(Zn)

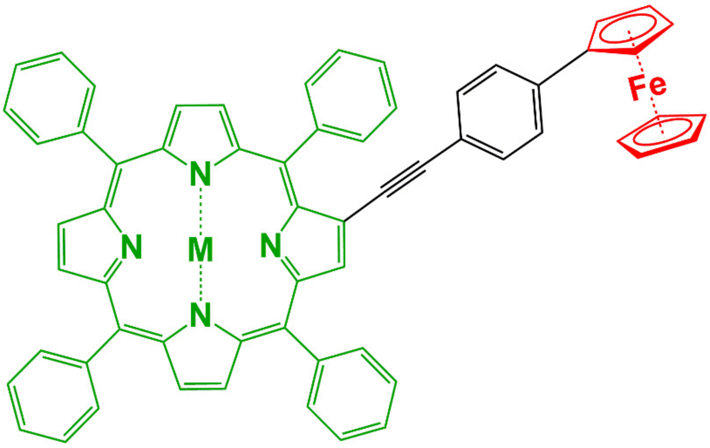

3b(Zn) M= Zn

^{1}H-NMR (300 MHz, $CDCl_3$): δ(ppm) 9.21(s, 1H), 8.84(m, 5H), 8.82(d, 2H), 8.75(d, 1H), 8.21(m, 8H), 7.75(m, 12H), 7.45(d, 2H), 4.72(s, 2H), 4.39(s, 2H), 4.10(s, 5H).

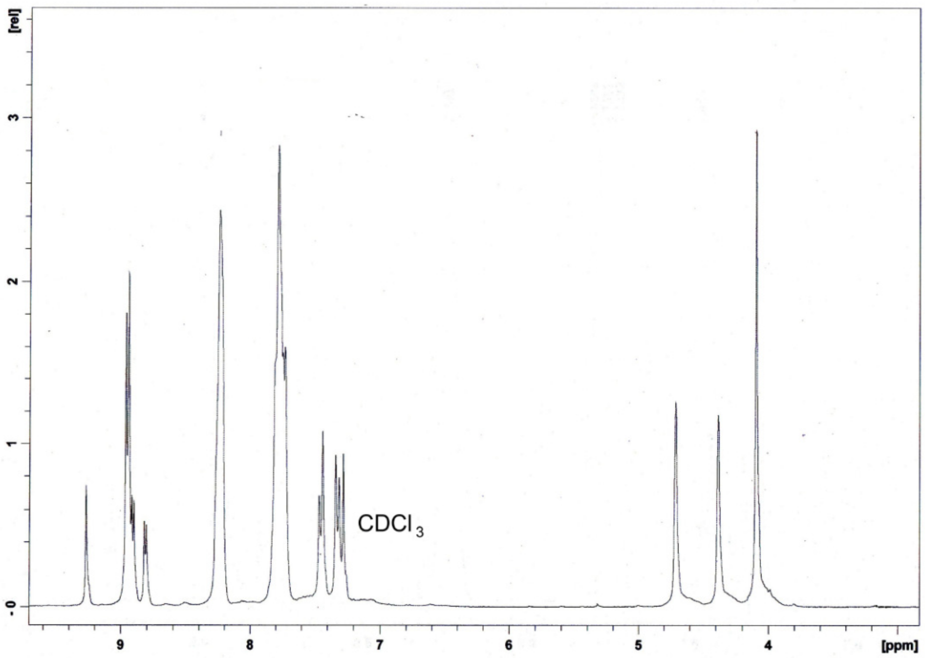

Figure S4. ^{1}H-NMR spectrum of compound **3b(Zn)** in $CDCl_3$ at 300 K at 400 MHz. .

MS(FAB+): *m*/*z* 963: $[M+H]^+$

Elemental Analysis

Anal. Calcd. for $C_{62}H_{40}N_4ZnFe$: C, 77.38; H, 4.18; N, 5.82. Found: C, 77.15; H, 4.17; N, 5.84

Compound 6-C60

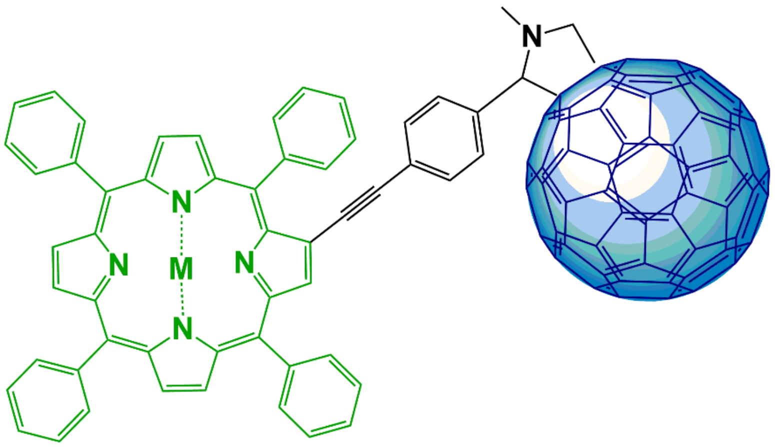

6-C60 **M= 2H**

^{1}H-NMR (400 MHz, $CDCl_3$): δ (ppm) 9.07 (s, 1H), 8.89 (s, 2H), 8.82 (d, 1H; J) 5.1 Hz), 8.79 (s,2H), 8.73 (d, 1H; J 5.1 Hz), 8.22 (br m, 5H), 7.79 (br m,15H), 7.61 (br, m, 2H), 7.43 (br, m, 2H), 4.96 (d, 1H; J) 9.2Hz), 4.89 (s, 1H), 4.22 (d, 1H; J) 9.2 Hz), 2.86 (s, 3H), -2.68 (s, 2H).

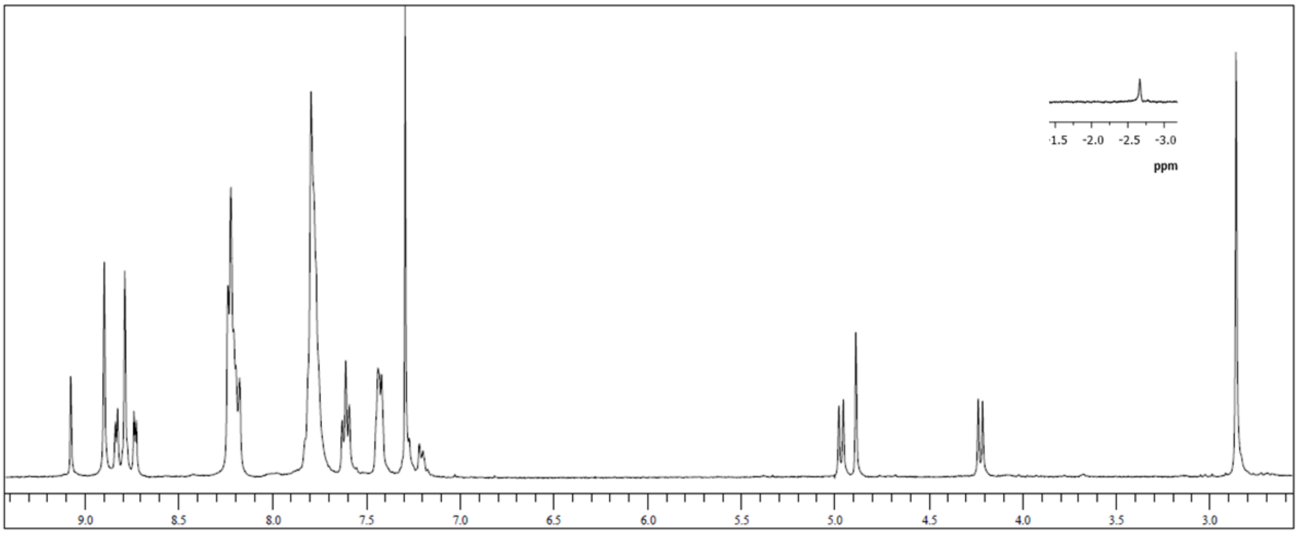

Figure S5. ^{1}H-NMR spectrum of compound **6-C60** in $CDCl_3$ at 300 K at 400 MHz.

MS(FAB+): *m/z* 1491 $[M + H]^+$, 769 $[M -720]^+$, 720 $[M - 769]^+$

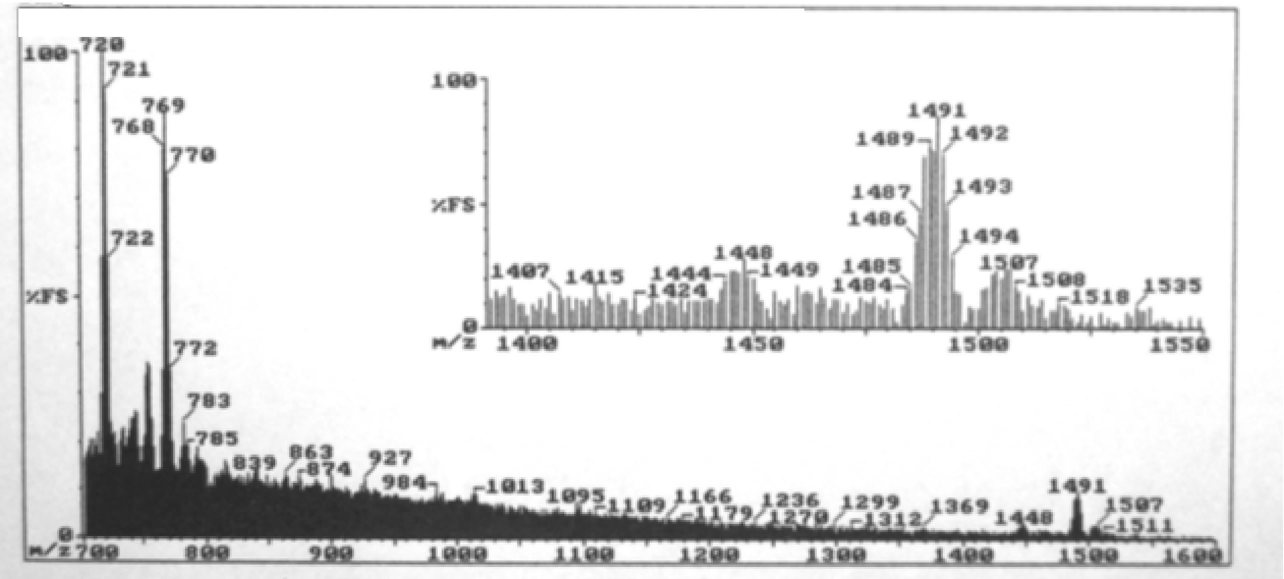

Figure S6. FAB MS spectrum of compound **6-C60** using as matrix NBA.

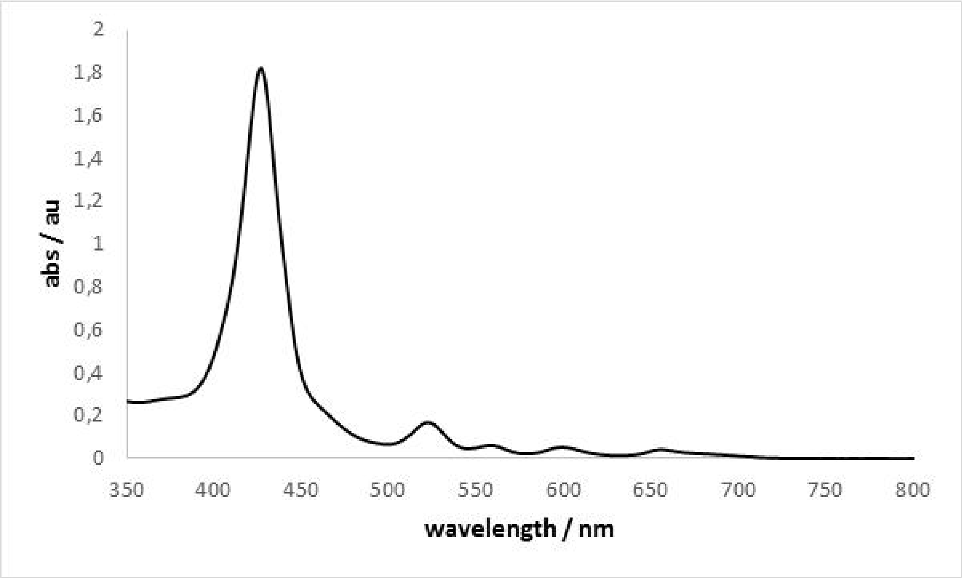

Figure S7. UV-Vis spectrum of compound **6-C60** in CH_2Cl_2.

Elemental Analysis

Anal. Calcd. for $C_{115}H_{40}N_5$ C, 92.60; H, 2.70; N, 4.70. Found: C, 98.85; H, 2.69; N, 4.72.

Compound 6(Zn)-C60

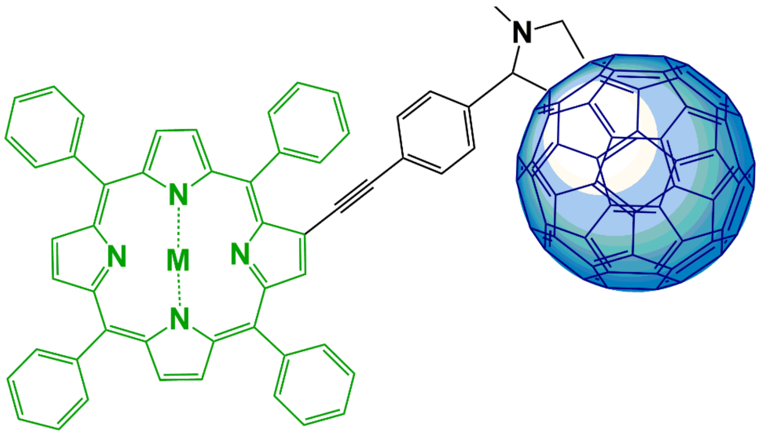

6(Zn)-C60 M= Zn

^{1}H-NMR (400 MHz, $CDCl_3$): δ(ppm) 9.24 (s, 1H,), 8.95 (s, 2H), 8.93 (s, 2H), 8.88 (d,1H; J=5.1 Hz), 8.77 (d, 1H; J=5.1 Hz), 8.22 (br m, 3H), 8.16 (br d, 2H; J=7.2 Hz), 7.80 (br m, 10H), 7.60 (m, 3H), 7.45-7.40 (br m, 4H), 7.20 (d, 2H; J=7.2 Hz), 4.95 (d, 1H; J=9.2 Hz), 4.85 (s, 1H), 4.20 (d, 1H; J=9.2 Hz), 2.87 (s, 3H).

MS (FAB+): *m/z:* 1552 $[M]^+$, 832 $[M-720]^+$, 720 $[M-832]^+$

Elemental Analysis

Anal. Calcd. for $C_{115}H_{38}N_5Zn$ C, 88.83; H, 2.46; N, 4.50, Zn, 4.21 Found: C, 88.57; H,2.45; N, 4.49.

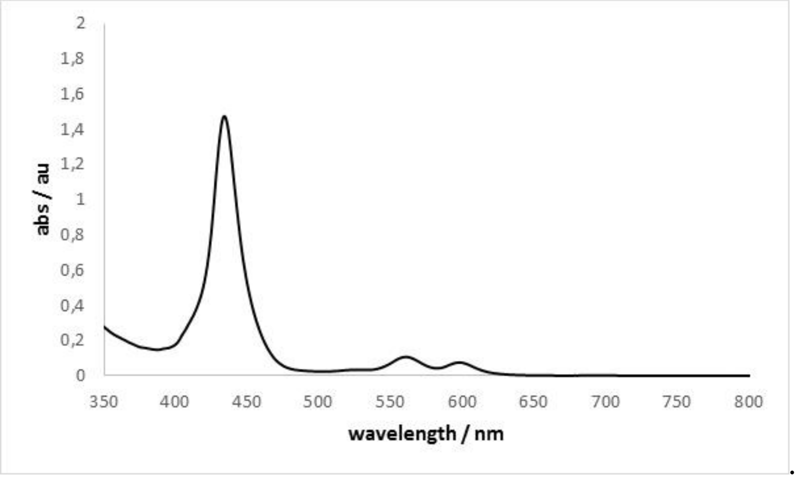

Figure S8. UV-Vis spectrum of compound **6(Zn)-C60** in CH_2Cl_2.

Compound 10a-C60

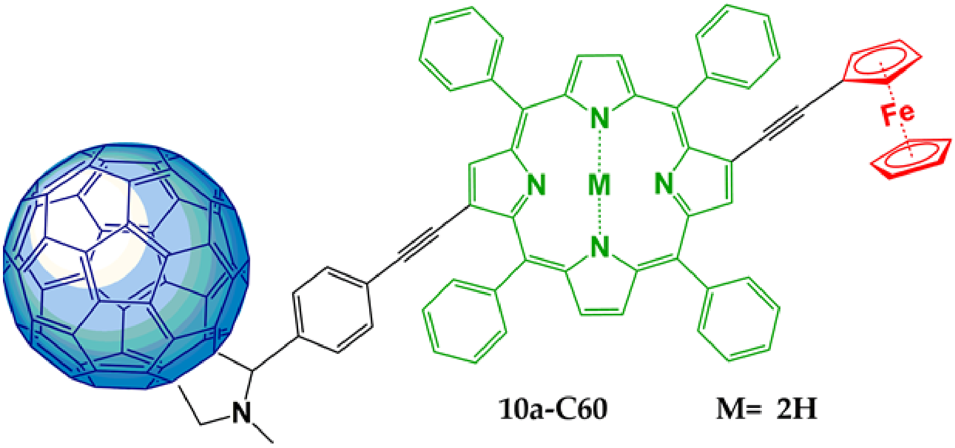

^{1}H-NMR (300 MHz, $CDCl_3$): δ (ppm) 9.04 (s, 1H), 8.99 (s,1H), 8.91 (t, 1H, J ¼ 2.9 Hz), 8.87 (t, 1H, 2.9 Hz), 8.84 (s, 1H), 8.78 (t,1H, J ¼ 5.9 Hz), 8.13 (m, 8H), 7.85 (m, 10H), 7.81 (m, 2H), 7.78 (m,2H), 4.77 (d, 1H, J ¼ 8.3 Hz), 4.65 (s, 1H), 4.36 (s, 3H), 4.24 (s, 6H),3.99 (d, 1H, J ¼ 8.3 Hz) 2.74 (s, 3H), -2.69 (s, 1H).

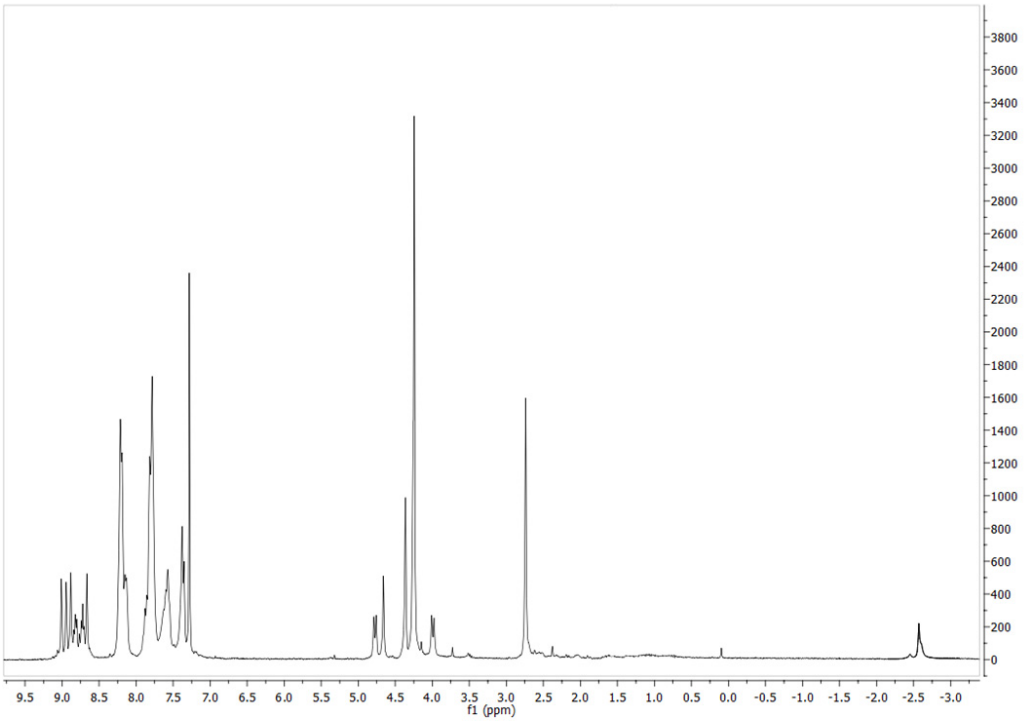

Figure S9. ^{1}H-NMR spectrum of compound **10a-C60** in $CDCl_3$ at 300 K at 400 MHz.

MS (MALDI-TOF): *m/z:* 1699.32 $[M + H]^+$

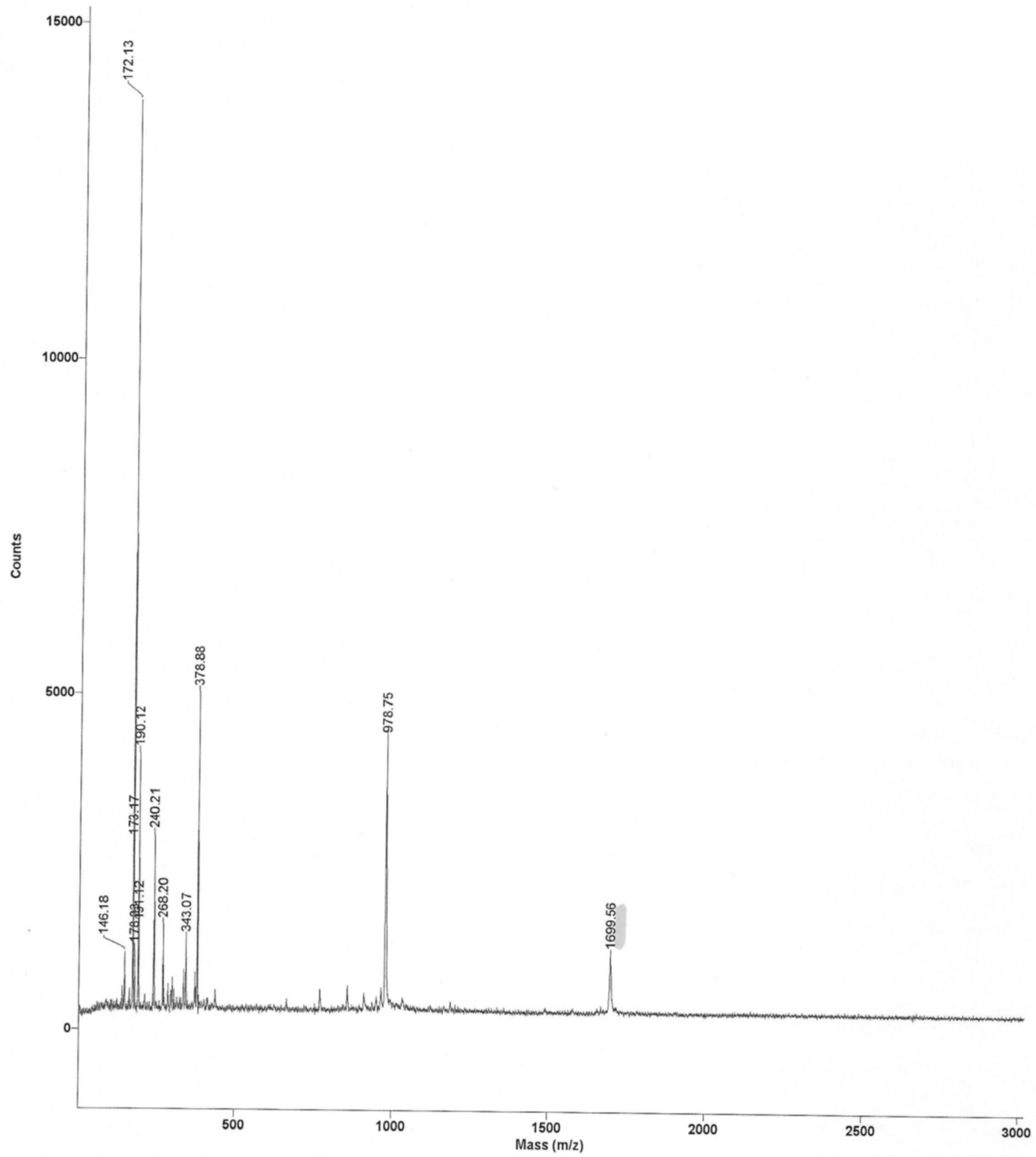

Figure S10. MALDI-TOF MS spectrum of compound **10a-C60** using as matrix gentisic acid.

Elemental Analysis

Anal. Calcd. for $C_{127}H_{47}N_5Fe$: C, 89.80; H, 2.78; N, 4.12. Found: C, 90.07; H, 2.79; N, 4.11.

Compound 10a(Zn)-C60

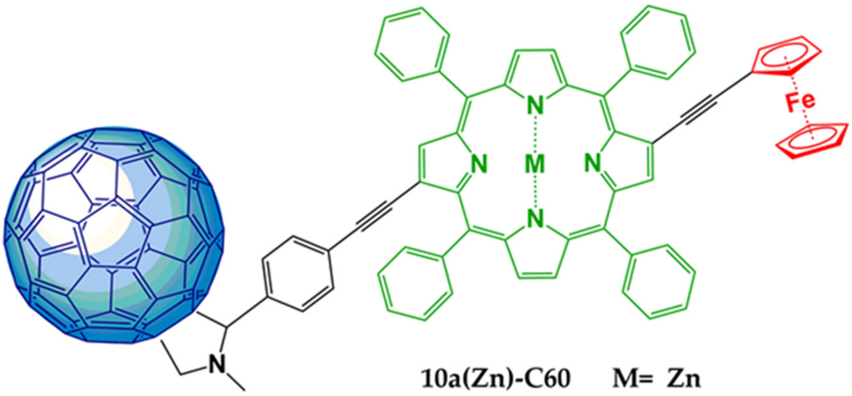

^{1}H-NMR (300 MHz, $CDCl_3$): δ (ppm) 9.18 (s, 1H), 9.12 (s,1H), 8.90 (m,2H), 8.82 (m, 1H), 8.67 (s, 1H), 8.18 (m, 8H), 7.82 (m, 12H), 7.57 (m,4H), 7.41 (d, 2H, J ¼ 9.2 Hz), 4.80 (d, 1H, J ¼ 9 Hz), 4.66 (s, 1H), 4.39 (s, 3H), 4.26 (s, 6H), 4.03 (d, 1H, J ¼ 9.2 Hz), 2.80 (s, 3H).

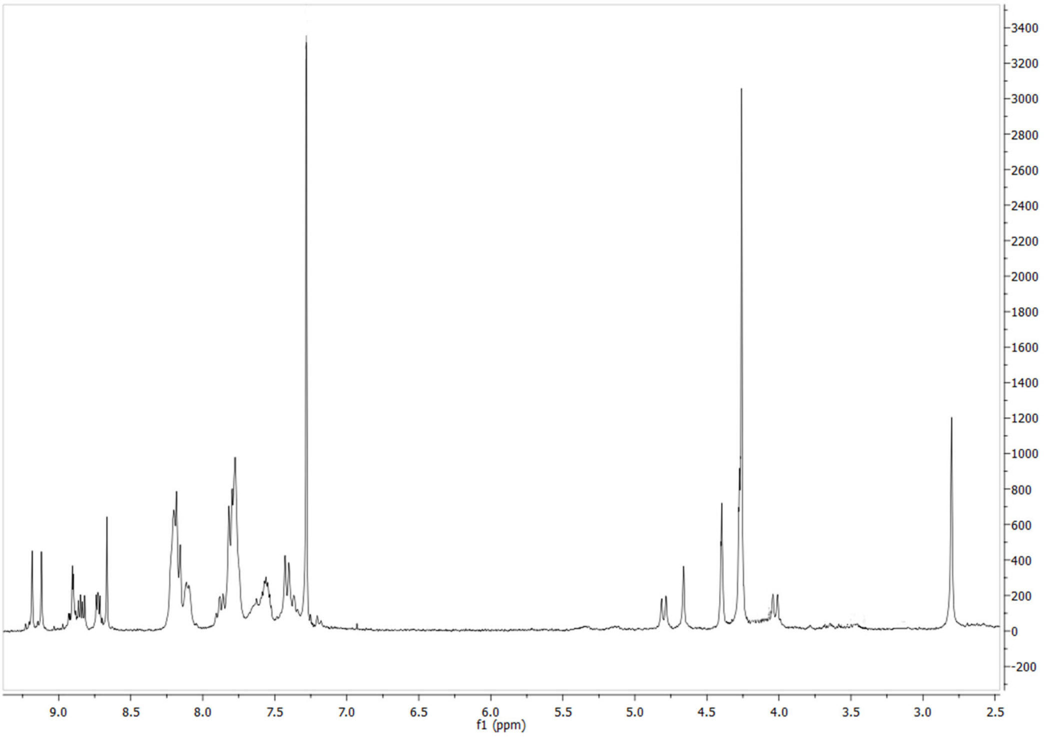

Figure S11. ^{1}H-NMR spectrum of compound **10a(Zn)-C60** in $CDCl_3$ at 300 K at 400 MHz. .

MS (MALDI-TOF): *m*/*z*: 1041.52 [M-C60]$^+$

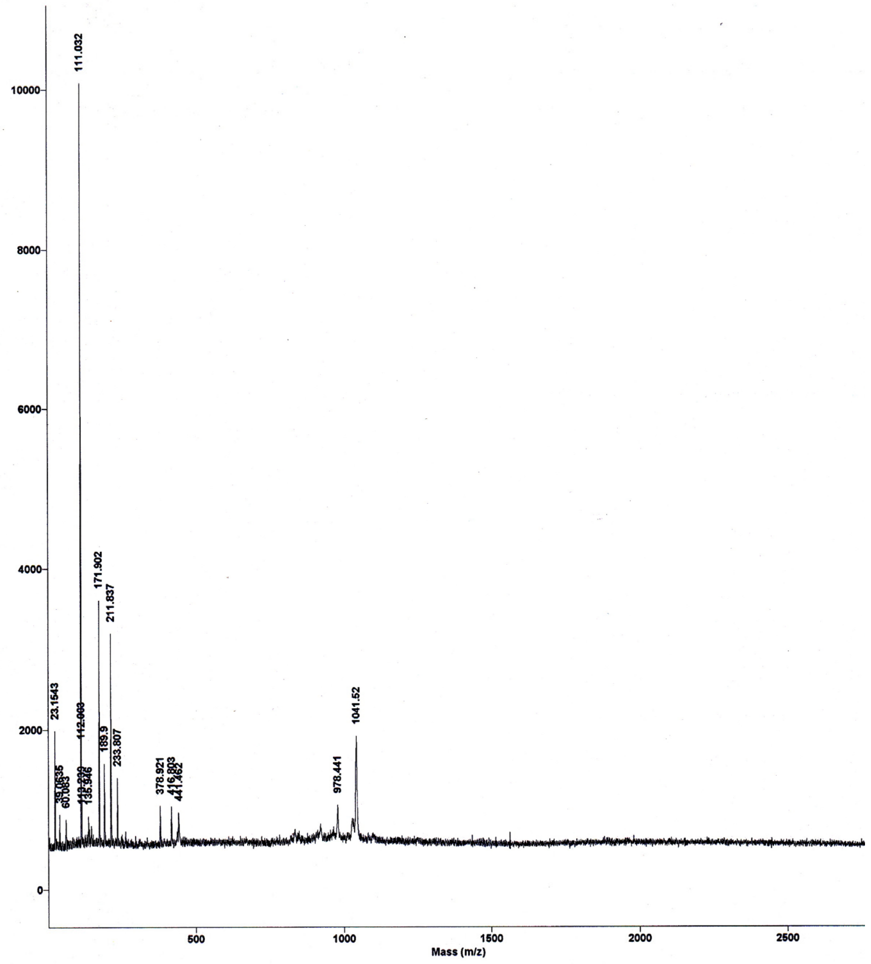

Figure S12. MALDI-TOF MS spectrum of compound **10a-C60** using as matrix gentisic acid.

Elemental Analysis

Anal. Calcd. for $C_{127}H_{45}N_5FeZn$:C, 86.57; H, 2.57; N, 3.97. Found: C, 86.83; H, 2.55; N, 3.99

Compound 10b-C60

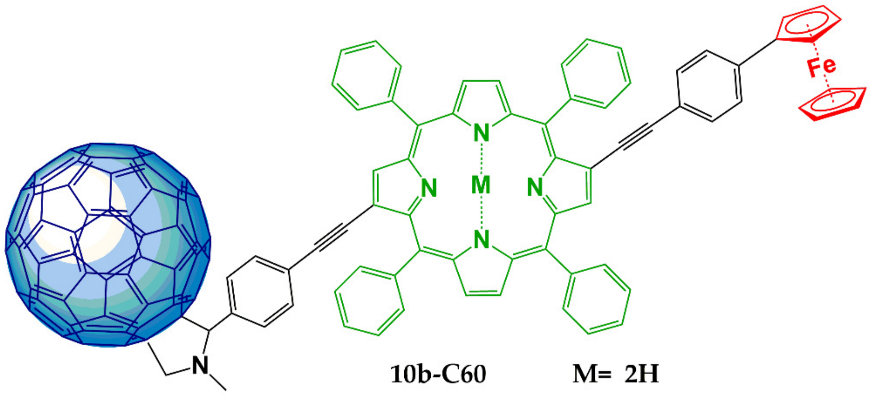

10b-C60 **M= 2H**

^{1}H-NMR (300 MHz, $CDCl_3$): δ (ppm) 9.02 (s,1H), 8.92 (d, 1H, J ¼ 1.9 Hz), 8.77 (m, 4H), 8.21 (m, 8H), 7.78 (m,12H), 7.59 (m, 2H), 7.47 (m, 6H), 4.90 (d, 1H, J ¼ 1.3 Hz), 4.81 (s, 1H), 4.70 (s, 2H), 4.38 (s, 2H), 4.14 (d, 1H, J ¼ 1.2 Hz) 4.08 (s, 5H), 2.81 (s,3H), -2.65 (s, 2H).

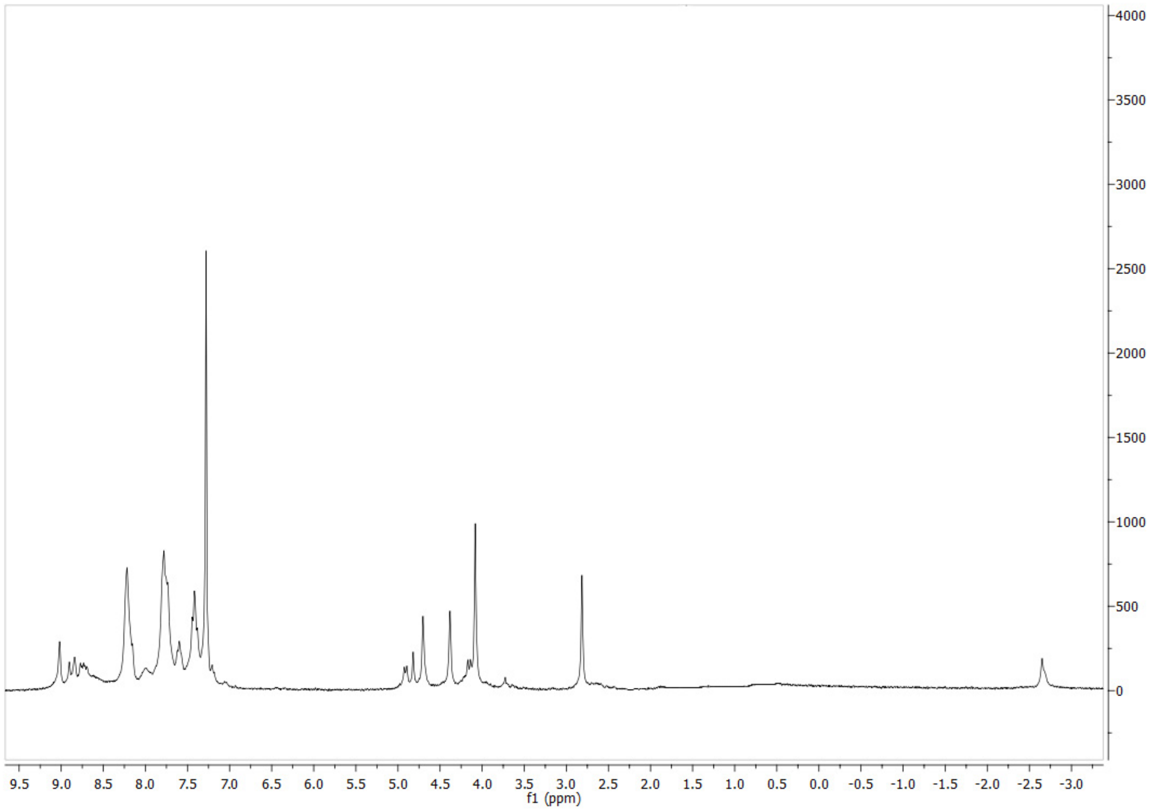

Figure S13. ^{1}H-NMR spectrum of compound **10b-C60** in $CDCl_3$ at 300 K at 400 MHz.

MS (MALDI-TOF): *m*/*z:* 1775.35 $[M + H]^+$,1055 $[M\text{-}C60]^+$

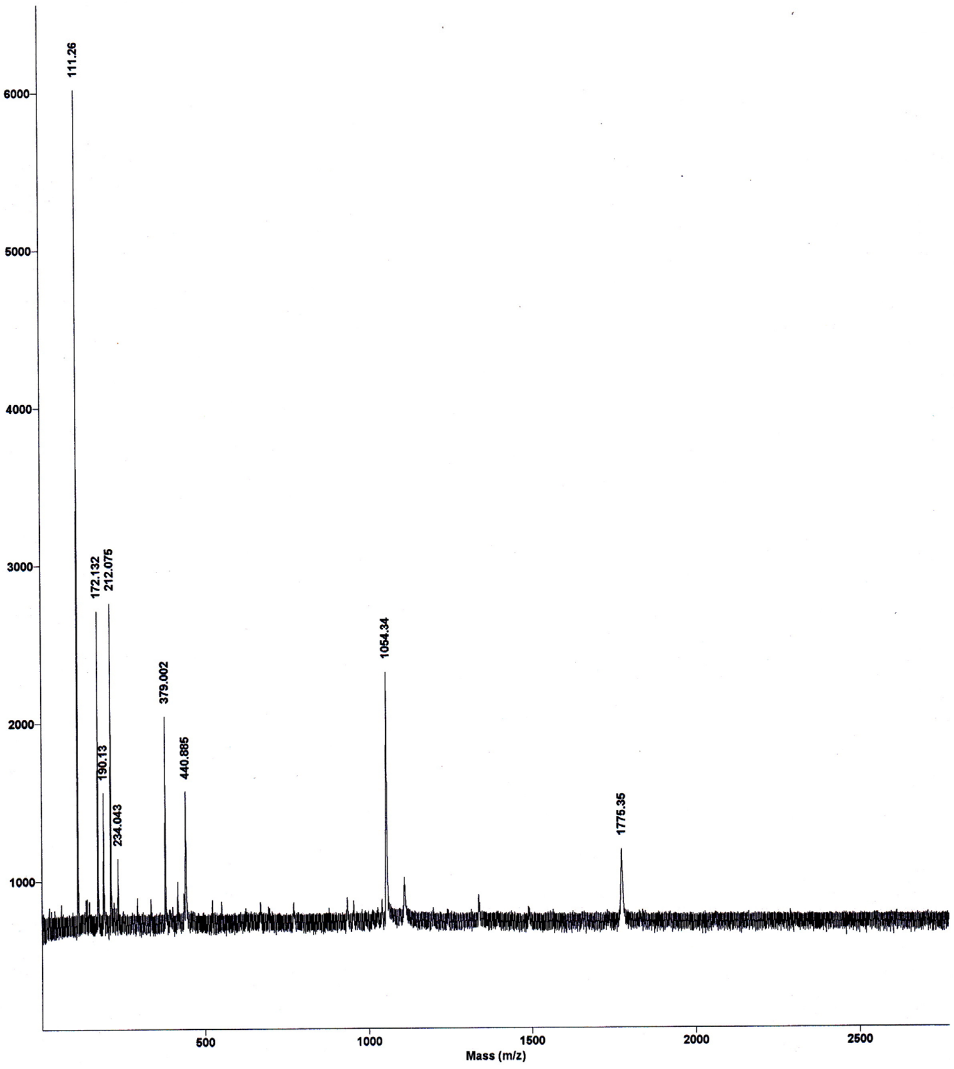

Figure S14. MALDI-TOF MS spectrum of compound **10b-C60** using as matrix gentisic acid.

Elemental Analysis

Anal. Calcd. for $C_{133}H_{51}N_5Fe$: C, 90.01; H, 2.89; N, 3.94. Found: C, 90.28; H, 2.88; N, 3.92.

Compound 10b(Zn)-C60

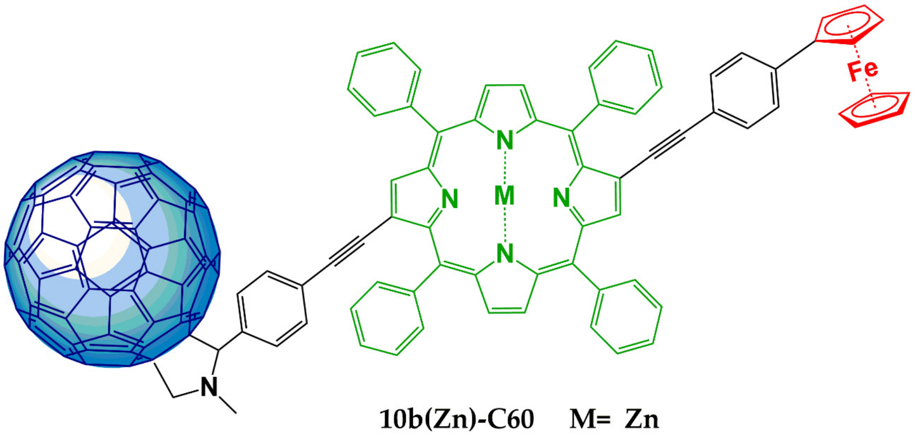

10b(Zn)-C60 M= Zn

^{1}H-NMR (300 MHz, $CDCl_3$): δ (ppm) 9.06 (s, 1H), 9.01 (s, 1H), 8.90 (m,2H), 8.82 (m, 1H), 8.67 (s, 1H), 8.21 (m, 8H), 7.78 (m, 12H), 7.57 (m,4H), 7.39 (m, 2H), 7.03 (d, 2H, J ¼ 6.2 Hz); 4.84 (d, 1H, J ¼ 9 Hz), 4.73 (s, 1H), 4.40 (s, 3H), 4.10 (s, 6H), 4.03 (d, 1H, J ¼ 9.2 Hz), 2.77 (s, 3H).

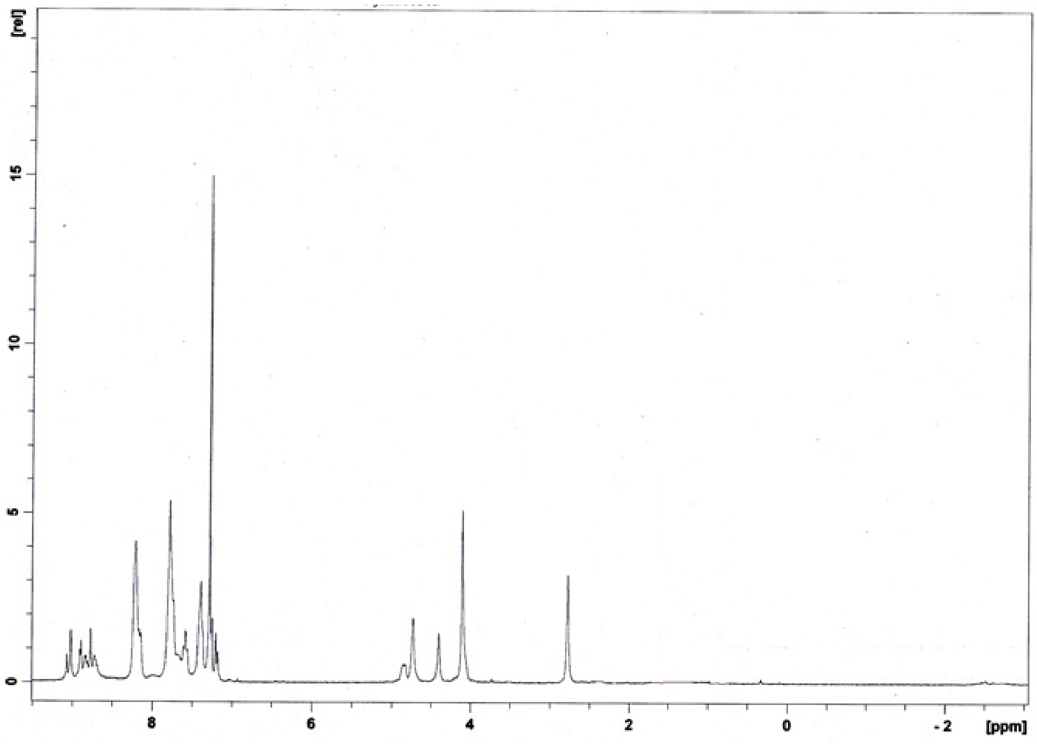

Figure S15. ^{1}H-NMR spectrum of compound **10b(Zn)-C60** in $CDCl_3$ at 300 K at 400 MHz.

MS (MALDI-TOF): *m*/*z*: 1117.05 [M-C60]$^+$

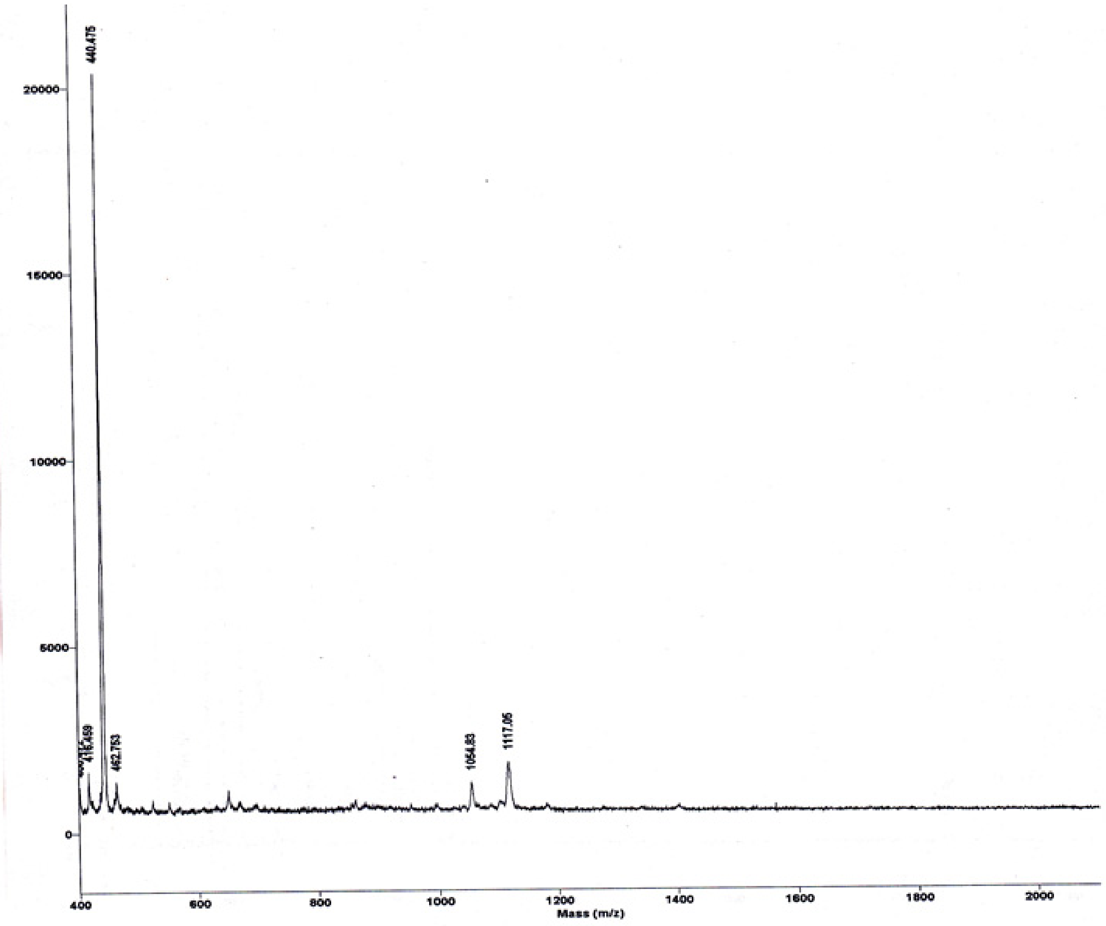

Figure S16. MALDI-TOF MS spectrum of compound **10b(Zn)-C60** using as matrix gentisic acid.

Elemental Analysis

Anal. Calcd. for $C_{133}H_{49}N_5FeZn$: C, 86.90; H, 2.68; N, 3.81. Found: C, 86.64; H, 2.67; N, 3.79.
